# Effectiveness of therapeutic patient education interventions for chronic diseases: A systematic review and meta-analyses of randomized controlled trials

**DOI:** 10.3389/fmed.2022.996528

**Published:** 2023-01-25

**Authors:** Jorge César Correia, Ahmed Waqas, Jean-Philippe Assal, Melanie J. Davies, Florence Somers, Alain Golay, Zoltan Pataky

**Affiliations:** ^1^Unit of Therapeutic Patient Education, Division of Endocrinology, Diabetology, Nutrition and Therapeutic Patient Education, World Health Organization Collaborating Centre, Geneva University Hospitals and University of Geneva, Geneva, Switzerland; ^2^Institute of Population Health, University of Liverpool, Liverpool, United Kingdom; ^3^Fondation recherche et formation pour l'enseignement du malade, Geneva, Switzerland; ^4^Diabetes Research Centre, National Institute for Health Research Leicester Biomedical Research Centre, Leicester, United Kingdom

**Keywords:** patient education, chronic disease, meta-analysis, chronic disorders, patient–centered care, health education and awareness, therapeutic patient education, patient empowerment

## Abstract

**Background:**

Chronic disorders are highly prevalent and are a major contributor to death and disability worldwide. Evidence has shown that therapeutic patient education (TPE) interventions are effective in improving a range of biomedical and psychological outcomes for a variety of chronic disorders. This has been demonstrated in scores of randomized controlled and evidence-synthesis studies. However, no quantitative evidence has been published so far on the content and effective teaching strategies in TPE programs. The present systematic review and meta-analysis aim to bridge this gap by answering the *who, what, and how* of TPE programs.

**Methods:**

Using a pretested search strategy, we searched the Web of Science, MEDLINE, CINAHL, PsycINFO, and the COCHRANE databases, from inception to August 2019. The search strategy was based on four comprehensive search concepts (patient education, chronic diseases, study design, and outcomes). After a careful screening for eligible studies, two reviewers extracted qualitative and quantitative data from the randomized controlled trials on the TPE interventions. We also developed a taxonomy of curriculum skills and intervention delivery techniques to aid the extraction of data in these domains.

**Results:**

We found that these interventions were effective in improving biological outcomes (SMD = 0.48; 95% CI: 0.38–0.57), adherence to the treatment regimen (SMD = 0.73; 95% CI: 0.46–1.002), knowledge (SMD = 1.22; 95% CI: 0.79–1.65), self-efficacy (SMD = 0.43; 95% CI: 0.30–0.56), and psychological health (SMD = −0.41; 95% CI: −0.53 to −0.29). This effectiveness was consistent across different delivery formats (individual, group, and electronic) and delivery agents (non-specialists vs. specialists).

**Conclusion:**

The flexibility in the choice of mode of delivery and curriculum development gives stakeholders an opportunity to scale up TPE interventions in healthcare settings.

**Systematic review registration:**

Identifier: CRD42019141294.

## Background

Chronic disorders are highly prevalent and are a major contributor to death and disability worldwide. In the United States, six out of every ten adults have a chronic disease, accounting for US$3.8 trillion in health costs every year (1). They are also the principal reason for seeking healthcare. In fact, more than 80% of all medical consultations globally concern chronic illnesses. Besides their adverse biomedical sequelae, these disorders are also associated with significant impairment in psychosocial functioning and poor quality of life (2).

Modern medical science has made significant strides in terms of both diagnostic tools and the treatment and management of chronic disorders. In the last decade, our understanding of the etiology of chronic diseases has significantly improved owing to advances in public health and epidemiology. This has brought to attention the adverse sequelae of unhealthy lifestyles and attitudes such as sedentariness, psychological stress, poor diet, tobacco use, and/or alcohol consumption (3, 4). The management of the chronic disease has also been improved by new technologies, drugs, and innovative procedures.

To achieve optimum care for these disorders, it is necessary to involve patients in their care as important stakeholders, decision-makers, and partners in the doctor–patient relationship (5). It is necessary to empower the patients with the necessary knowledge, skills, and understanding of their disease processes, so they can effectively manage their condition by collaborating with their health caregivers. The successful management of chronic disorders requires that the chronically ill patient be actively involved in the follow-up process of his/her disease. As a result, the patient is placed at the center of the patient–healthcare professional relationship and the biomedical care process.

Therapeutic patient education (TPE) delivered by healthcare professionals aims to empower patients to understand, be involved in the clinical decision-making process, and effectively manage their conditions (6, 7). Several patient education interventions have been shown to improve medical outcomes, self-efficacy, and satisfaction with treatment among patients with different chronic conditions (5, 8, 9). These interventions guide lifestyle changes and help to improve adherence to treatment (5, 8, 9). These skills are important to build self-efficacy and self-care behaviors in patients to improve biomedical and psychosocial outcomes in chronic disorders (10, 11).

The potential of these interventions has been recognized by several stakeholders worldwide, making them a cornerstone of promotion, prevention, and treatment guidelines around the globe (8, 12–14). The field of patient education is a highly interdisciplinary area (15). Moreover, heterogeneous strategies and technologies are utilized for the delivery of patient education. For instance, Friedman et al. presented evidence from 23 systematic reviews regarding a variety of teaching techniques, including computer technology, audiovisual aids, written materials, and demonstrations to be successful in improving knowledge and satisfaction and decreasing anxiety and depression among patients with chronic disorders. However, they emphasized the importance of culturally acceptable methods in designing TPE programs (16).

To date, there is a lack of comprehensive evidence about the effectiveness of TPE programs in improving health outcomes in chronic disorders. Moreover, no quantitative evidence has been published so far on the development of interventions and effective teaching strategies in TPE programs. The present systematic review and meta-analysis aim to bridge this gap by answering the *who, what, and how* of TPE programs specifically for secondary and tertiary prevention and treatment of chronic disorders by exploring the following questions:

a) What is the effectiveness of TPE programs in improving health outcomes in chronic disorders?
b) Who are the most effective delivery agents of TPE programs?
c) What delivery techniques are most effective in the delivery of TPE programs?

This review is part of a larger project, Putting the pAtient fiRsT: maNagemEnt of chRonic diSeases by tHerapeutIc Patient education (PARTNERSHIP), which is leading a series of evidence-synthesis studies on the role of therapeutic patient education in the management of chronic disorders.

## Methods

This systematic review and meta-analysis were conducted in accordance with the PRISMA guidelines (17). Prior to the performance of review processes, the protocol was registered in PROSPERO (CRD42019141294) (18). Using a pretested search strategy, we searched the Web of Science, MEDLINE, CINAHL, PsycINFO, and the COCHRANE databases from inception to August 2019. The search strategy was based on four comprehensive search concepts (patient education, chronic diseases, study design, and outcomes) presented in Table 1. Articles published only in English and French were included, with no restrictions applied to the region or publication year of studies.

**Table 1:** Search strategy adapted for PubMed database.

**Concept**	**Search terms**
Chronic diseases	(chronic[ti/ab] OR “chronic disease”[ti/ab] OR long-term[ti/ab] OR “chronic disease”[MeSH] OR “chronic disease hospital”[MeSH]) AND (respiratory[ti/ab] OR pulmonary[ti/ab] OR kidney[ti/ab] OR cerebrovascular[ti/ab] OR infection[ti/ab] OR cancer[ti/ab] OR metabolic[ti/ab] OR gastr^*^[ti/ab] OR cardiac[ti/ab] OR hypertension[ti/ab] OR asthma[ti/ab] OR COPD[ti/ab] OR neuro^*^[ti/ab] OR coronary[ti/ab] OR diabetes[ti/ab] OR hypertensive[ti/ab] OR urinary[ti/ab] OR urological[ti/ab] OR reproductive[ti/ab] OR cardiovascular[ti/ab] OR skin[ti/ab] OR dermatolog^*^[ti/ab] OR psychiatr^*^[ti/ab] OR mental[ti/ab] OR joint[ti/ab] OR hormon^*^[ti/ab] OR “heart disease”[ti/ab] OR disease^*^[ti/ab] OR endocrin^*^[ti/ab] OR neoplas^*^[ti/ab] OR communicable[ti/ab] OR non-communicable[ti/ab])
Outcome	(“Treatment outcome”[ti/ab] OR outcome[ti/ab] OR psychosocial[ti/ab] OR lab^*^[ti/ab] OR “physical outcome”[ti/ab] OR stress[ti/ab] OR depress^*^[ti/ab] OR “disease recurrence”[ti/ab] OR perception[ti/ab] OR “Disease progression”[ti/ab] OR “self-care”[ti/ab] OR complication^*^[ti/ab] OR hospitalization[ti/ab] OR self-efficacy[ti/ab] OR “self-management”[ti/ab] OR compliance[ti/ab] OR adherence[ti/ab] OR knowledge[ti/ab] OR attitude[ti/ab] OR behavior[ti/ab] OR “quality of life”[ti/ab])
Intervention	(“Health education” [MeSH] OR “patient education”[MeSH] OR “Health education” [ti/ab] OR “patient education”[ti/ab] OR psychoeducation[ti/ab] OR “therapeutic education”[ti/ab] OR “consumer health information” OR “health knowledge”[ti/ab] OR “client education”[ti/ab])
Study design	(trial^*^[ti/ab] OR RCT[ti/ab] OR randomized-controlled[ti/ab] OR “cluster randomized controlled”[ti/ab] OR intervention[ti/ab] OR “clinical trial“[PT] OR “controlled clinical trial”[PT])

This review focuses on the TPE programs specifically for secondary and tertiary prevention and treatment of chronic disorders across all medical specialties. For this review, we defined TPE interventions as disease-specific educational interventions for patients with chronic disorders, delivered by specialists or trained non-specialist healthcare professionals, aimed at improving their competency to effectively manage their illnesses through mobilization and use of different resources. These resources include but are not limited to knowledge, self-management, and other cognitive strategies as well as social, emotional, and experiential elements (19).

Two reviewers working independently from one another screened titles and abstracts of bibliographic records retrieved from electronic searching. Articles deemed eligible for inclusion will be scrutinized further as per the inclusion and exclusion criteria by reviewing the full texts. If the decisions of reviewers about the inclusion of studies differed at either of the two stages, a senior author arbitrated the process. We included all those randomized and cluster-randomized controlled trials that tested therapeutic education interventions conducted among adults (aged 18 years or older) suffering from any type of chronic disease. We excluded non-randomized or quasi-experimental studies with pre-post designs, short-form of publications, and overlapping datasets.

Data extraction was done using an excel-based pre-tested proforma by two reviewers. Prior to actual data extraction, each reviewer independently extracted 10% of the studies, which were then reviewed to establish inter-rater reliability. In any study, only primary outcomes tested at primary time points were included. We considered a range of outcomes for inclusion, including any biological parameters, psychological symptomology and quality of life indicators, self-efficacy, compliance, adherence to treatment, and health knowledge, attitudes, and behaviors. Among moderators, we included variables pertaining to the design of interventions, contents and elements, the dosage of intervention, and the type of delivery agents.

A pretested taxonomy was developed for content covered in TPE interventions after reviewing key papers in the field of patient education and behavior change techniques (20). The following five major categories of content covered in TPE interventions were identified: understanding disease processes, disease management, lifestyle changes, cognitive and behavioral coping, and interpersonal skills. This taxonomy was further divided into 22 different skills (Supplementary Table 1). The category of understanding disease processes was broken down into the etiology of the disease, behavior related to health and illness, and treatment modalities. Disease management included disease management, self-monitoring, adapting drug doses and initiating self-treatment, the performance of technical gestures, and dealing with problems caused by illnesses. Lifestyle changes included implementing lifestyle changes, being aware of health-related risk factors, and preventing avoidable complications. Cognitive and behavioral coping included self-confidence and self-awareness, stress management, critical thinking, decision-making and problem-solving, goal setting, strengthening oneself, self-care, and coping mechanisms. Finally, interpersonal skills included organizational information, communication skills, and social support.

For meta-analyses, we extracted means and standard deviations for quantitative outcomes, and for binary outcomes, frequencies and sample sizes were extracted (21). Due to expected heterogeneity in the reporting of these trials, we used random effects analyses for pooling data for all outcomes. Heterogeneity was considered significant at *I*^2^ > 60% for the review (21). Sensitivity analyses were conducted to assess the contribution of outliers to effect sizes. Publication bias was assessed by visualizing Begg's funnel plot along with Egger's regression statistic (22). In cases of significant publication bias, the trim and fill method (with random effects) was used to impute the missing studies (23). Subgroup analyses with mixed effects were run to check the effectiveness of this intervention across different delivery agents. Meta-regression analyses were used to assess the association of different teaching methods and content of the intervention, with the effect size for each outcome. Since there was considerable heterogeneity in the reporting of biomedical outcomes, we also used full random effects analyses to combine studies within each subgroup (24). A random effects model is used to combine subgroups and yield the overall effect. The study-to-study variance (tau-squared) is assumed to be the same for all subgroups—this value is computed within subgroups and then pooled across subgroups (25).

The Cochrane tool for the assessment of the risk of bias in RCTs was used to rate the risk of bias across the following six domains: random sequence generation, allocation concealment, blinding of outcome assessors, other sources of biases, attrition bias, and selective reporting (26). We did not rate bias in blinding procedures for participants and personnel, because it is not possible to do so in patient education interventions.

It was not appropriate or possible to involve patients or the public in the design, conduct, report, or disseminate plans of our research.

## Results

**Figure 1:**
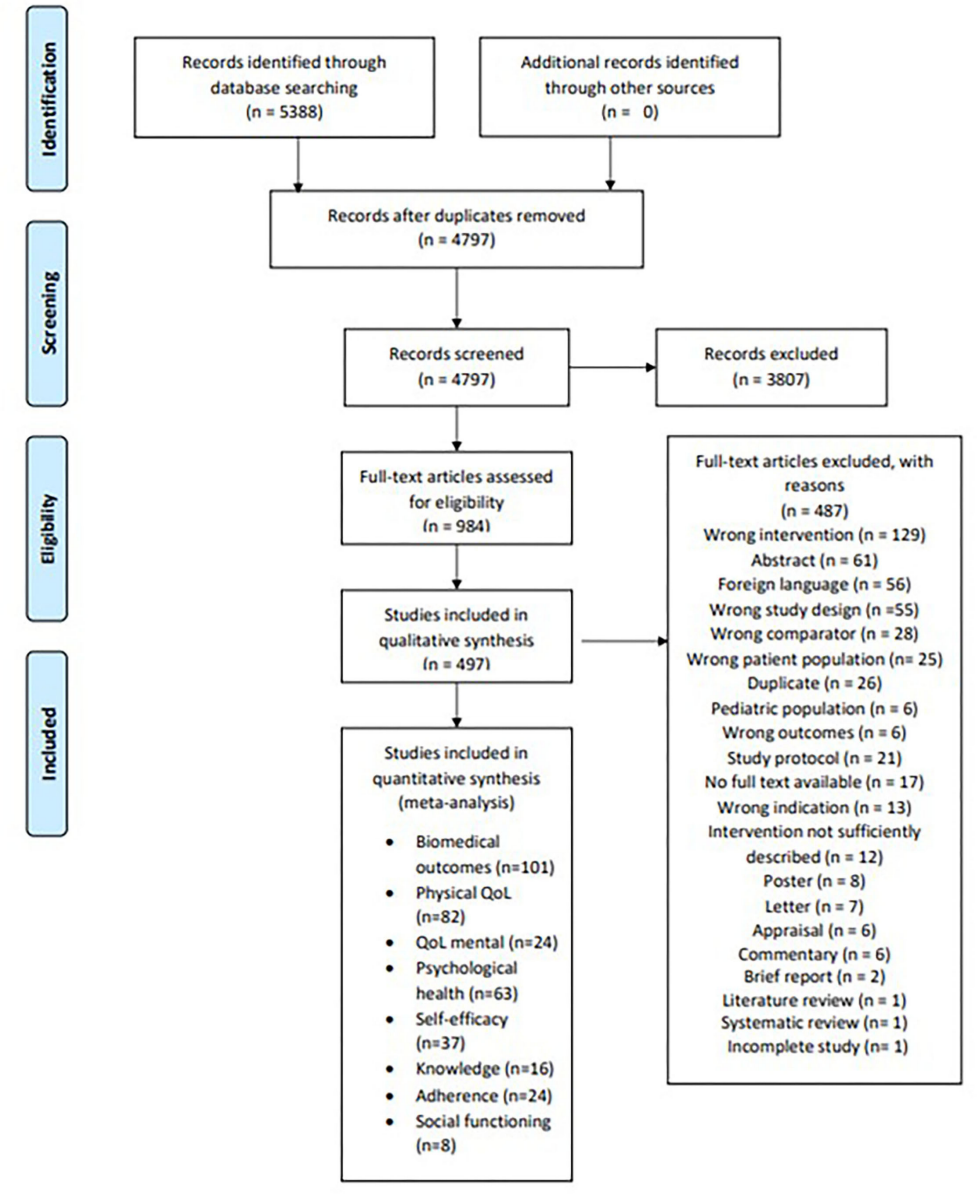
PRISMA flowchart demonstrating the process of the screening process of studies.

Our database search yielded a total of 5,388 titles and abstracts, out of which 1,365 duplicates were deleted. A total of 4,797 titles and abstracts were then reviewed against our eligibility criteria, out of which 984 full texts were included. Major reasons for the exclusion of full texts were interventions lacking an educational approach (*n* = 129), conference abstracts (*n* = 61), and languages other than English (*n* = 56). The search process yielded a total of 497 full texts eligible for inclusion (Figure 1). The earliest trial was published in 1980, and six trials were published from 1980 to 1990. From 1991 to 2000, a total of 30 trials were published, followed by 173 from 2000 to 2010 and 288 from 2011 to 2019.

**Figure 2:**
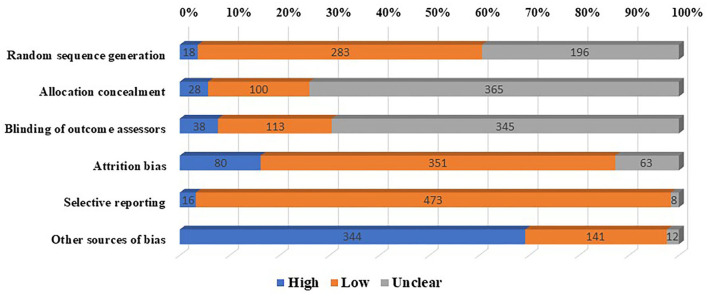
Risk of bias among included trials of therapeutic patient education interventions.

As per the Cochrane tool for the assessment of the risk of bias in RCTs, higher or unclear risk of bias was seen across allocation concealment (*n* = 397), blinding of outcome assessors (*n* = 384), and other sources of bias (*n* = 356). Low risk of bias was more frequently reported across random sequence generation, selective reporting, and attrition bias (Figure 2, Supplementary material 1).

These 497 trials spanned multiple medical specialties. These patient education interventions were most frequently studied in respiratory disorders (*n* = 82), followed by cardiovascular disorders (*n* = 74), metabolic disorders (*n* = 55), musculoskeletal disorders (*n* = 41), psychiatric disorders (*n* = 45), and oncology (*n* = 37). Among specific diseases, the most frequently targeted disorders were chronic obstructive pulmonary disease (*n* = 61), diabetes mellitus (*n* = 50), chronic heart diseases (*n* = 38), chronic kidney disease, and end-stage renal disease (*n* = 36), hypertension (*n* = 19), breast cancer (*n* = 19), chronic pain (*n* = 22), asthma (*n* = 16), depression (*n* = 12), schizophrenia (*n* = 11), and stroke (*n* = 10) (Supplementary material 1).

These interventions were most frequently delivered by utilizing allied healthcare providers (*n* = 272), followed by multidisciplinary teams comprising a combination of specialists and non-specialists (*n* = 125), research teams (*n* = 48), doctors only (*n* = 25), peer and peer leaders (*n* = 23), and self-help-oriented Internet media (*n* = 4). A total of 167 of these interventions were delivered face to face in groups (*n* = 167), individuals (*n* = 149), telephones (*n* = 33), and Internet (*n* = 19), while 129 interventions used multiple modalities (Supplementary material 1).

**Figure 3:**
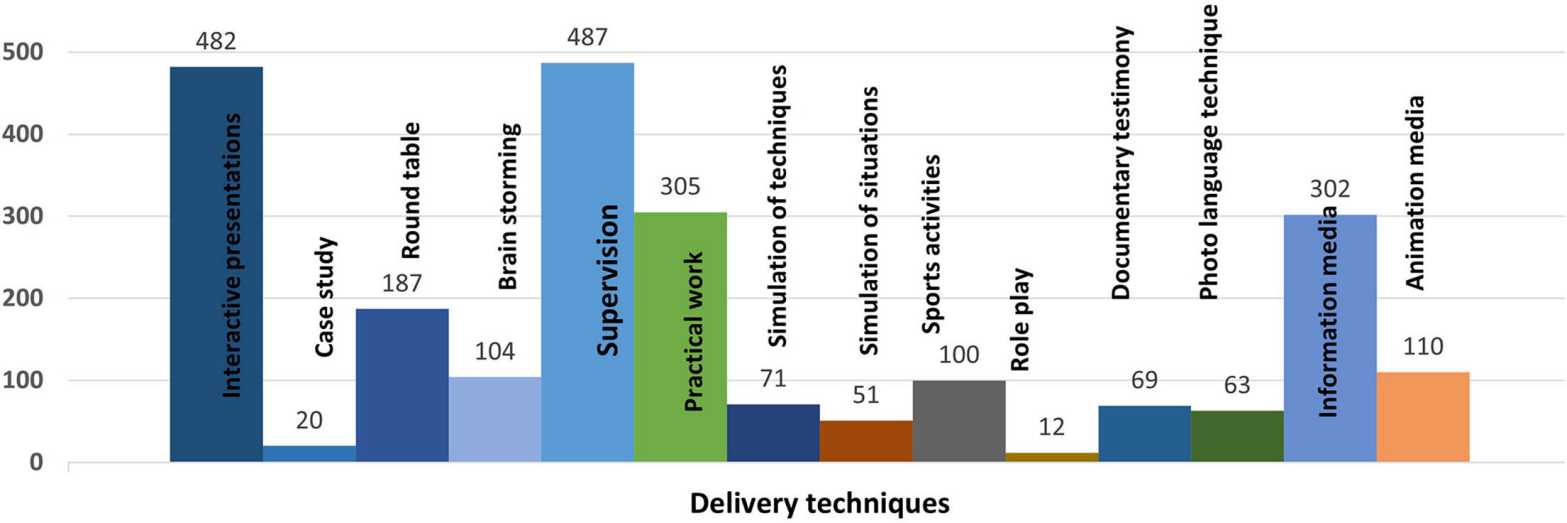
Proportion of studies employing specific techniques for delivery of therapeutic patient education interventions.

Several strategies were employed for the delivery of these interventions; nonetheless, most employed strategies were didactic, including interactive presentations (*n* = 482), the use of information media, such as brochures and pamphlets (*n* = 302), roundtable discussions (*n* = 187), brainstorming sessions (*n* = 104), and documentary testimonies (*n* = 69). Many interventions employed practical demonstrations by using case studies (*n* = 20), simulations of situations (*n* = 51) and gestures and techniques (*n* = 71), practical work (*n* = 305), and supervision from experts (*n* = 487). More active forms of teaching included sports activities (*n* =100), role plays (*n* = 12), and photo-language techniques (*n* = 63). A total of 110 of these interventions also included animation media, such as cartoons and animations (*n* = 110) (Figure 3 and Supplementary material 1).

**Figure 4:**
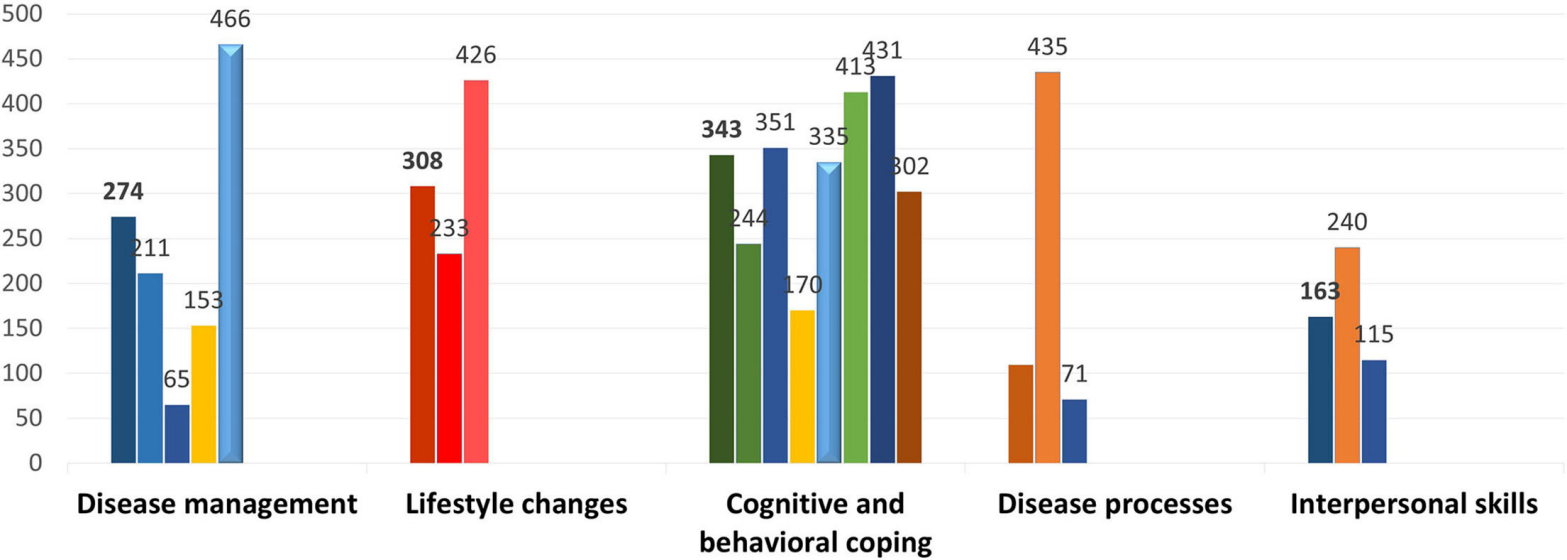
Strategies and skills taught as part of therapeutic patient education interventions.

The content of the interventions spanned across the following five cross-cutting themes (Figure 4): disease management (*n* = 479), lifestyle changes (*n* = 459), cognitive and behavioral coping (*n* = 494), understanding disease processes (*n* = 456), and interpersonal skills (*n* = 336). The types of delivery agents across different interventions did not differ in the use of disease management strategies (*F* = 0.59, *p* = 0.71), lifestyle changes (*F* = 1.08, *p* = 0.37), disease processes (*F* = 1.13, *p* = 0.35), and interpersonal skills (*F* = 2.02, *p* = 0.07). Only the use of behavioral and cognitive coping skills yielded statistical significance (*F* = 2.43, *p* = 0.03), with interventions delivered by peers and peer leaders employing these skills most frequently.

The curriculum of interventions differed according to their delivery formats. The use of lifestyle change strategies was more evident among interventions delivered through mixed and individual formats [*χ*^2^_(4)_ = 10.81, *p* = 0.03], coping skills in group formats [*χ*^2^_(4)_ = 31.48, *p* < 0.001], disease processes in group and mixed formats [*χ*^2^_(4)_ = 31.32, *p* = 0.01], and teaching interpersonal skills was more evident among interventions delivered through the Internet and group formats [$\chi_{\left(4\right)}^{2}$ = 30.02, *p* < 0.001; Supplementary Figures 1–5]. There were no significant associations between the type of delivery agent and different strategies employed among interventions, except for the use of cognitive, and behavioral coping skills. These skills were most frequently used by peers and peer leaders while delivering the TPE interventions [*F*_(5)_ = 2.43, *p* = 0.03; Supplementary Figure 1].

### Effectiveness and moderator analyses

#### Biological indicators

Biological outcomes were reported in a total of 101 trials, with a cumulative sample size of 27,293 participants. The time point of reporting these outcomes ranged from post-intervention to 120 months. There was substantial heterogeneity in the reporting of these outcomes (*I*^2^ = 90.31%, *X*^2^ = 1,034.86, *p* < 0.001). Random effects analyses revealed a moderate effect size in favor of the intervention group (SMD = 0.329; 95% CI: 0.24–0.41). There was significant evidence for publication bias (Egger's regression *p* = 0.008). The effect size increased slightly after adjusting for publication bias. After the imputation of 25 studies to the right of the mean, the effect size increased to 0.48 (95% CI: 0.38–0.57). No significant subgroup differences in effect sizes were found across different delivery formats (*P* = 0.54); however, significant differences were evident across different interventions, delivery agents (*P* < 0.001), and disorders (*P* = 0.02). None of the elements were significantly associated with effect sizes (*R*^2^ = 1%, *p* = 0.89).

Detailed results for fully random effects analyses for biomedical outcomes are provided in Figure 5, fully random effects analyses for all biopsychosocial outcomes are provided in Figure 6, detailed forest plots for individual outcomes are provided in Supplementary Figures 6–13, and funnel plots are provided in Supplementary Figures 14–20. Detailed subgroup analyses for all outcomes have been provided in Supplementary Tables 1–8.

**Figure 5:**
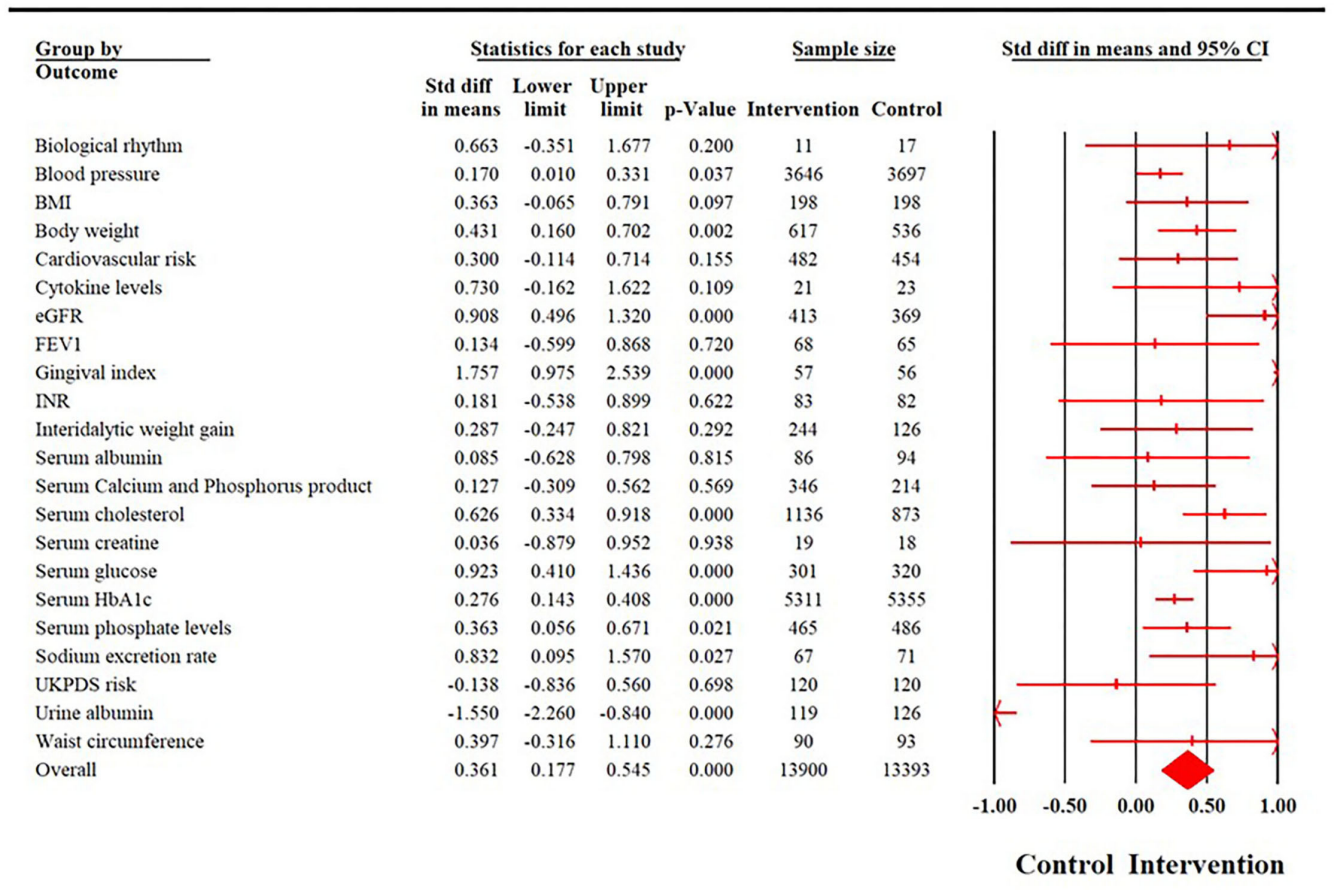
Summary of a forest plot demonstrating the effectiveness of therapeutic patient education interventions among different biological outcomes.

**Figure 6:**
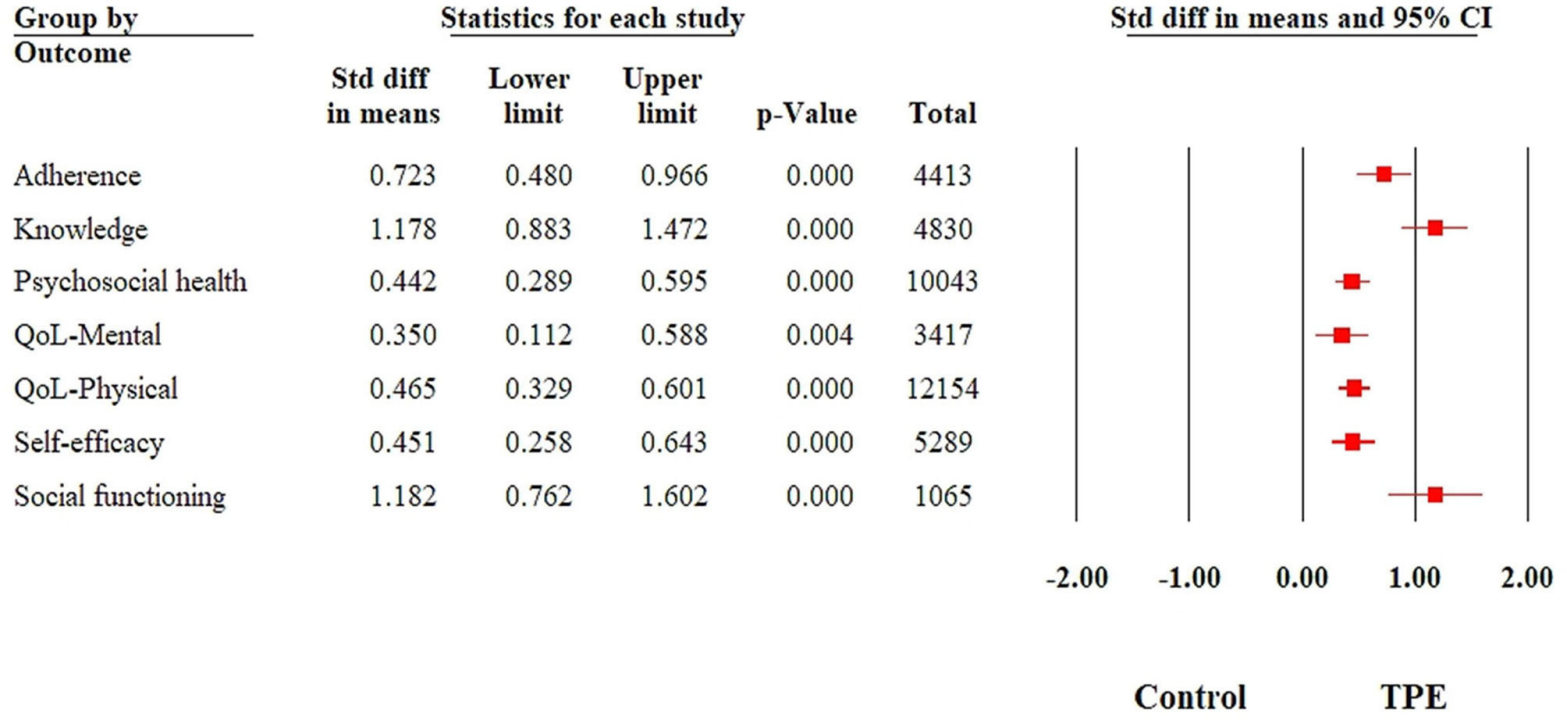
Summary of a forest plot demonstrating the effectiveness of therapeutic patient education interventions among different psychosocial and psychological outcomes.

#### Adherence

Adherence was reported in 24 studies with a cumulative sample size of 4,413 participants. The time point of reporting these outcomes ranged from post-intervention to 9 months of follow-up. There was substantial heterogeneity in the reporting of these outcomes (*I*^2^ = 91.07%, *p* < 0.001). Random effects analyses revealed strong effect sizes in favor of the intervention group (SMD = 0.73; 95% CI: 0.46–1.002) (Figure 6). No publication bias was revealed in the reporting of this outcome (Egger's regression *P* = 0.55).

No significant associations of effect size for adherence were found with disorder type (*Q* = 16.2, *p* = 0.13), delivery agents (*Q* = 0.64, *p* = 0.89), and format of delivery (*Q* = 0.82, *p* = 0.94). The type of intervention elements yielded no significant relationships with effect size (*R*^2^ analog = 0.35).

#### Knowledge

Health-related knowledge was reported in 16 studies, representing a cumulative sample size of 4,830 participants. There was substantial heterogeneity in the reporting of this outcome (*I*^2^ = 97.42%, *Q* = 581.19, *p* < 0.001). The intervention group undergoing TPE interventions reported large gains in health knowledge (SMD = 1.22; 95% CI: 0.79–1.65) (Figure 6). Sensitivity analyses did not reveal any significant changes in effect size. There was no evidence of publication bias (Egger's regression *P* = 0.90). None of the intervention strategies were associated with the effect size (*R*^2^ analog = 0.35). No significant associations were found with the type of delivery agent (*Q* = 1.87, *p* = 0.60), the format of delivery (*Q* = 6.92, *p* = 0.14), or the type of disorders.

#### Self-efficacy

Self-efficacy was reported in a total of 37 studies, with a cumulative sample size of 5,289 participants. Time points ranged from post-intervention to 12 months. There was substantial heterogeneity in the reporting of this outcome (*I*^2^ = 79.59%, *Q* = 176.41, *p* < 0.001). A moderate effect size was noted in favor of the intervention group (SMD = 0.43; 95% CI: 0.30–0.56) (Figure 6). No changes in statistical significance were noted in sensitivity analyses. The significant publication was evident (Egger's regression *P* = 0.03). A total of 10 studies to the right of the mean were adjusted using random effects, which increased the effect size (SMD= 0.60, 95% CI: 0.45–0.74).

Meta-regression analyses explained 26% of the variance in heterogeneity in self-efficacy, where the use of interpersonal skills was associated with higher gains in self-efficacy (B = 0.26, SE = 0.13, *p* = 0.05). Other strategies were not associated with effect sizes. No significant associations were noted with types of disorders (*Q* = 12.04, *p* = 0.21) and delivery agents (*Q* = 1.21, *p* = 0.75). The format of delivery was significantly associated with self-efficacy (*Q* = 7.82, *p* = 0.05), where interventions delivered by telephones (SMD = 0.92, 95% CI: 0.52–1.33) revealed the highest effect sizes (albeit imprecise), followed by those delivered in groups (SMD = 0.45, 95% CI: 0.26–0.63).

#### Psychological health

Psychological outcomes were reported in 63 studies, with a cumulative sample size of 10,043 participants. Time points for reporting these outcomes ranged from post-intervention to 2 years post-intervention. There was substantial heterogeneity in the reporting of these outcomes (*I*^2^ = 88.35%, *Q* = 506.46, *p* < 0.001). Moderate effect sizes were evidently in favor of the intervention group (SMD = −0.43; 95% CI: −0.55 to −0.31) (Figure 6). No changes were evident in the statistical significance of this outcome by performing sensitivity analyses. Publication bias was evident (Egger's regression *p* = 0.02), which upon adjustment of 13 studies to the left of the mean led to an increase in effect size (SMD = −0.57, 95% CI: −0.69 to −0.44). Meta-regression with intervention strategies as covariates explained only 10% of the variance, with none of the strategies achieving significance. The highest effect sizes were notable for interventions delivered by allied health workers and doctors (*Q* = 20.75, *p* < 0.001). No significant subgroup differences were noted for the types of disorders (*Q* = 7.4, *p* = 0.76) and format of delivery (*Q* = 2.6, *p* = 0.62).

#### QoL-Mental

Mental health-related quality of life was reported in a total of 24 studies, with a cumulative sample size of 3,417 study participants. The time point for reporting these outcomes ranged from post-intervention to 19 months. There was evidence for substantial statistical heterogeneity (*I*^2^ = 93.34%, *Q* = 348.31, *p* < 0.001). Random effect analyses revealed weak to moderate effect sizes in favor of the intervention group (SMD = 0.35; 95% CI: 0.08–0.63) (Figure 6). Egger's regression test revealed no evidence of publication bias (*P* = 0.18); however, the funnel plot revealed significant asymmetry. Duval and Tweedie's trim and fill method was used to impute 7 studies to the right of the mean, leading to a higher SMD (0.66; 95% CI: 0.35–0.97).

Meta-regression accounted for only 10% of the variance in QoL-mental; none of the strategies were associated with effect sizes. Subgroup analyses revealed significant subgroup differences according to types of disorders (*Q* = 18.94, *p* = 0.02) and types of delivery agents (*Q* = 11.65, *p* = 0.02), where allied healthcare workers and research teams brought about the greatest yield in QoL-mental. The format of delivery was not associated with any subgroup differences (*Q* = 1.63, *p* = 0.65).

#### QoL-Physical

Physical health-related quality of life was reported in 75 studies (*n* = 12,154), with time points ranging from post-intervention to 24 months follow-up. There was substantial heterogeneity (*I*^2^ = 90.85%, *Q* = 808.6, *p* < 0.001). Moderate strength effect sizes were noted in favor of the intervention group (SMD = 0.46; 95% CI: 0.33–0.59) (Figure 6). There was significant evidence for publication bias (Egger's regression *p* = 0.008). Duval and Tweedie trim and fill method were used to impute 15 studies to the right of the mean, leading to an improvement in effect size (SMD = 0.66; 95% CI: 0.52–0.81).

Meta-regression with different intervention elements explained 7% of the variance in heterogeneity in this outcome, where imparting interpersonal skills was associated with higher effect sizes (B = 0.25, SE = 0.12, *p* = 0.03). Subgroup analyses revealed significant differences in effect sizes across different disorders (*Q* = 81.67, *p* < 0.001) and delivery agents (*Q* = 23.75, *p* < 0.001). Allied health workers, research staff, and multidisciplinary teams yielded higher effect sizes than their counterparts. Allied health worker-delivered interventions led to the highest improvement in physical health-related QoL.

#### Social functioning

Social functioning was presented as a primary outcome in eight trials, with a cumulative sample size of 1,065 trial participants. The time point reporting these outcomes ranged from post-intervention to 6 months follow-up. There was substantial heterogeneity in the reporting of this outcome (*I*^2^ = 97.24%, *X*^2^ = 253.38, *p* < 0.001). Random effects revealed a very high effect size in favor of the intervention group (SMD = 1.258; 95% CI: 0.40–2.11) (Figure 6). Sensitivity analyses did not reveal any changes in the statistical significance of this outcome. There was evidence of significant publication bias (Egger's regression *P* = 0.06). Publication bias adjusted point estimates revealed a higher effect size (SMD = 1.68; 95% CI: 0.66–2.71). Subgroup analyses did not reveal any differences in effect sizes among different delivery agents (*Q* = 4.02, *p*= 0.26) and formats of delivery (*Q* = 1.00, *p* = 0.8).

## Discussion

The present study synthesizes information on the effectiveness of TPE interventions across different specialties of medicine. We showed that these interventions were effective across all chronic disorders, leading to improvements in biomedical and the quality of life and psychosocial outcomes. A total of 497 randomized controlled trials were reviewed to generate a large-scale systematic review of evidence. We found that the TPE interventions yield excellent benefits in improving a range of outcomes among patients with chronic disorders. Moderate to large improvements are seen in biomedical outcomes, psychological health, and psychosocial functioning. The large volume of included studies allowed us to undertake complex subgroup and meta-regression analyses to compare different formats of delivery of these interventions and the types of delivery agents involved. We were also able to delineate the taxonomy, types of techniques, and curriculum components employed in the development of TPE interventions. We found that TPE interventions yield benefits for patients with chronic disorders when delivered using different strategies and formats.

In this large-scale systematic review, we found that TPE interventions yielded small to moderate strength effect sizes across a range of biomedical and psychosocial outcomes. The effectiveness of these TPE interventions has been demonstrated in a plethora of experimental and observational studies (27, 28). The TPE interventions work through increasing knowledge of the disorder, prevention, and medication, as well as the practical skills needed for the self-management of chronic disorders (27, 28). This has also been demonstrated in the present meta-analyses, where moderate to large improvements were seen in these outcomes among patients undergoing TPE interventions. An improvement in knowledge regarding the disorders also leads to better adherence to treatment regimens and the adoption of a healthy lifestyle (29, 30). An improvement in these outcomes translates to better biological outcomes and improvement in the severity of chronic disorders, as demonstrated in this review, where small to moderate improvements were noted among the intervention group (SMD = 0.48; 95% CI: 0.38–0.57).

The present systematic review revealed that most of the tested interventions utilized didactic techniques for the delivery of TPE interventions. Although such TPE interventions may be less interactive, they are more resource-effective than personalized one-to-one interventions for a large-scale implementation (31, 32). This is particularly important in regions where stakeholders may look for a balance between the clinical and cost-effectiveness of TPE interventions. Our analyses also highlight that the intervention *dosage*, i.e., effective delivery of TPE interventions, is more important than the *regimen*, i.e., using different curriculum techniques. As evident in the meta-regression analyses, using heterogeneous curriculum or skills, as noted in our taxonomy of intervention elements, did not account for heterogeneity in the effect sizes. This highlights that all elements of interventions and their combinations are equally effective to some extent.

Among the different delivery agents delivering the TPE interventions, research teams often yielded better effect sizes. This is perhaps because the research teams are better trained to maintain fidelity and competency in a research setting. However, this is not possible in pragmatic and real-world settings. A higher proportion of these interventions were delivered by either multidisciplinary teams or non-specialist healthcare workers. We found that interventions delivered by non-specialists are as effective as those delivered by healthcare professionals or multidisciplinary teams. This task-sharing approach is effective for delivery for a range of disorders, including physical and mental health conditions (33, 34). Furthermore, the delivery of group-based and face-to-face interventions was also similarly effective for a range of disorders, thus giving the stakeholders an opportunity to tailor the successful implementation of TPE interventions in healthcare and community settings.

The present systematic review and meta-analyses have several strengths. First, it is the first large-scale meta-analysis that synthesizes evidence for TPE interventions across all clinical specialties. This extensive work led to the curation of 497 RCTs, offering the opportunity to conduct meaningful interventional and patient-level subgroup analyses. Our findings demonstrate that TPE interventions are effective across all clinical specialties in improving biomedical and psychological outcomes. It presents opportunities for large-scale implementation by virtue of flexibility in the use of lay health workers and specialist health workers; delivery using different formats; and flexibility in the curriculum development of these interventions. Although this systematic review offers an up-to-date exercise in evidence synthesis about TPE interventions, we did not evaluate their cost-effectiveness. We encourage future investigators to work on this aspect. A limitation of this review was our inability to conduct subgroup analyses for using different didactic and interactive delivery of TPE interventions. This was due to the extent of overlap and multicollinearity in these variables. We encourage future authors to conduct dimension reduction analyses to alleviate these issues before using meta-regression analyses to elucidate these aspects of TPE.

In conclusion, TPE interventions are an excellent resource for improving care for patients with chronic disorders. These interventions work across a range of contexts and delivery formats and thus can be tailored to different health settings as per available resources. Allied healthcare staff could be an excellent resource to be mobilized for the delivery of TPE to achieve optimum patient care without burdening physicians.

## Data availability statement

The original contributions presented in the study are included in the article/Supplementary material, further inquiries can be directed to the corresponding author.

## Author contributions

JC, AW, AG, and ZP conceptualized the study, drafted the protocol for the systematic review, and meta-analysis. JC and FS searched the academic databases and identified the eligible trials. JC and AW extracted the data and wrote the initial draft of the manuscript. AW conducted the meta-analysis. JC, AW, and ZP interpreted the results. ZP, FS, AG, MD, and J-PA conducted critical review of the manuscript. All authors approved the final version of the manuscript for submission.
