# Supplementary material for: Effectiveness of therapeutic patient education interventions for chronic diseases: A systematic review and meta-analyses of randomized controlled trials
Source: Front Med (Lausanne). 2023 Jan 25;9:996528. doi: 10.3389/fmed.2022.996528 (PMC9905441; doi:10.3389/fmed.2022.996528)
Supplement: Supplementary file 1 [file Data_Sheet_1.docx]

**Supplementary material**

**Citation**

Correia JC, Waqas A, Assal J-P, Davies MJ, Somers F, Golay A and Pataky Z (2023). Effectiveness of therapeutic patient education interventions for chronic diseases: A systematic review and meta-analyses of randomized controlled trials. Front. Med. 9:996528. doi: 10.3389/fmed.2022.996528

| **Content** | **Page** |
| --- | --- |
| Supplementary table 1: Taxonomy of strategies explored in therapeutic patient education interventions | 3 |
| Supplementary figure 1: Means plot for delivery agents vs behavioral and cognitive coping skills | 4 |
| Supplementary figure 2:Use of lifestyle changes related strategies across different modes of delivery of interventions | 5 |
| Supplementary figure 3: Use of coping related strategies across different modes of delivery of interventions | 6 |
| Supplementary figure 4: Use of disease process related strategies across different modes of delivery of interventions | 7 |
| Supplementary figure 5: Use of interpersonal skills related strategies across different modes of delivery of interventions | 8 |
| Supplementary Figure 6: Forest plot for social functioning | 9 |
| Supplementary Figure 7: Forest plot for adherence to treatment regimen | 10 |
| Supplementary Figure 8: Forest plot for illness related knowledge | 11 |
| Supplementary Figure 9: Forest plot for Self-efficacy | 12 |
| Supplementary Figure 10: Forest plot for psychological health | 13 |
| Supplementary Figure 11: Forest plot for quality of life- mental domain | 14 |
| Supplementary Figure 12: Forest plot for physical health related quality of life | 15 |
| Supplementary Figure 13: Forest plot for biomedical outcomes | 16 |
| Supplementary figure 14: Funnel plot for social functioning | 18 |
| Supplementary figure 15: Funnel plot for adherence to treatment regimen | 19 |
| Supplementary figure 16: Funnel plot for health related knowledge | 19 |
| Supplementary figure 17: Funnel plot for self-efficacy | 20 |
| Supplementary figure 18: Funnel plot for psychological health | 21 |
| Supplementary figure 19: Funnel plot for mental health related quality of life | 22 |
| Supplementary figure 20: Funnel plot for physical health related quality of life | 23 |
| Supplementary table 1: Moderator analyses for outcome of social functioning | 24 |
| Supplementary table 2: Moderator analyses for outcome of adherence | 25 |
| Supplementary table 3: Moderator analyses for outcome of health knowledge | 26 |
| Supplementary table 4: Moderator analyses for outcome of self-efficacy | 27 |
| Supplementary table 5: Moderator analyses for outcome of psychological health | 28 |
| Supplementary table 6: Moderator analyses for outcome of QoL-mental | 29 |
| Supplementary table 7: Moderator analyses for outcome of QoL-physical health | 30 |
| Supplementary table 8: Moderator analyses for outcome of biomedical indicators | 31 |

Supplementary table 1: Taxonomy of strategies explored in therapeutic patient education interventions

| **Category** | **Content** | **Frequency** |
| --- | --- | --- |
| Disease management | Relieve symptoms | 274 |
|  | Self-monitoring | 211 |
|  | Adapt drug doses, and initiate self-treatment | 65 |
|  | Perform technical gestures and care | 163 |
|  | Deal with problems caused by illness | 466 |
| Lifestyle changes | Implement lifestyle changes | 308 |
|  | Awareness of health-related risk factors | 233 |
|  | Preventing avoidable complications | 426 |
| Cognitive & behavioural coping | Self-awareness | 343 |
|  | Stress management | 244 |
|  | Critical thinking | 351 |
|  | Problem solving | 170 |
|  | Set goals and make choices | 335 |
|  | Observe, assess and strengthen yourself | 413 |
|  | Self-care | 431 |
|  | Coping mechanisms | 302 |
| Disease processes | Etiology of the disease | 109 |
|  | Behaviour related to health and illness | 435 |
|  | Treatment modalities | 71 |
| Interpersonal skills | Organizational information | 163 |
|  | Communication skills | 240 |
|  | Identifying and inciting social support | 116 |


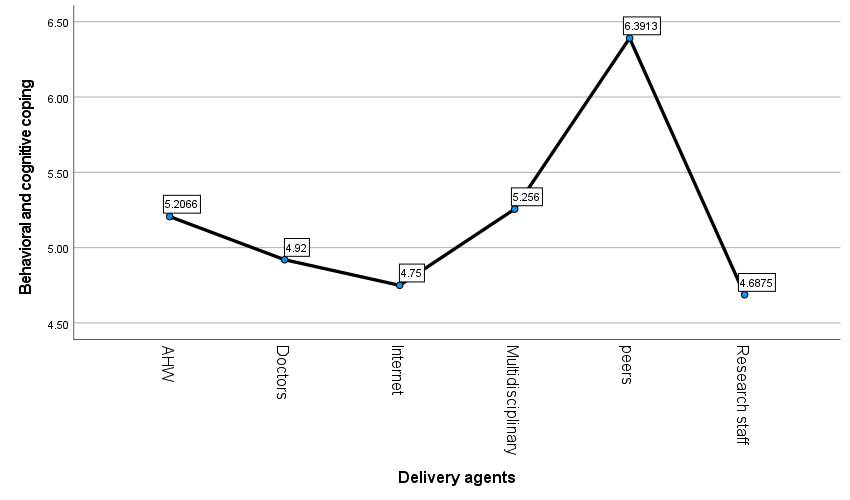


Supplementary figure 1: Means plot for delivery agents vs behavioral and cognitive coping skills


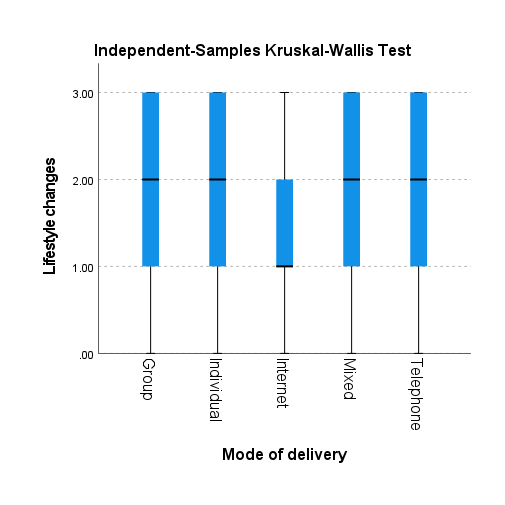


Supplementary figure 2:Use of lifestyle changes related strategies across different modes of delivery of interventions


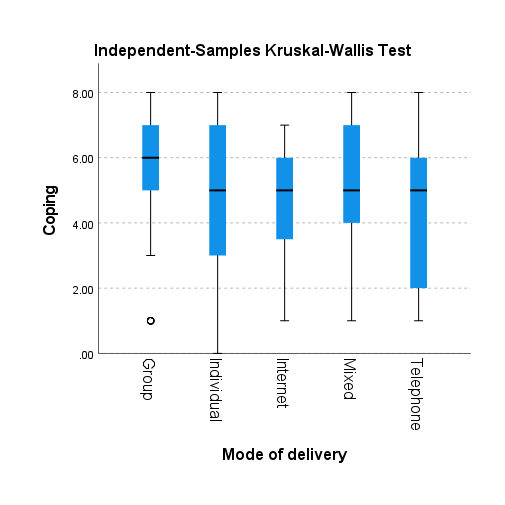


Supplementary figure 3: Use of coping related strategies across different modes of delivery of interventions


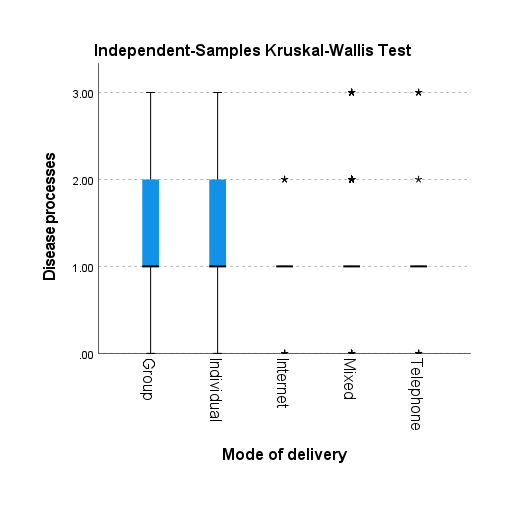


Supplementary figure 4: Use of disease process related strategies across different modes of delivery of interventions


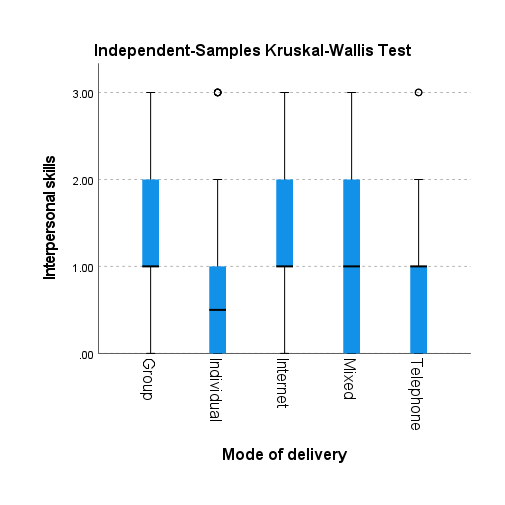


Supplementary figure 5: Use of interpersonal skills related strategies across different modes of delivery of interventions

Supplementary Figure 6: Forest plot for social functioning

Supplementary Figure 7: Forest plot for adherence to treatment regimen

Supplementary Figure 8: Forest plot for illness related knowledge

Supplementary Figure 9: Forest plot for Self-efficacy

Supplementary Figure 10: Forest plot for psychological health


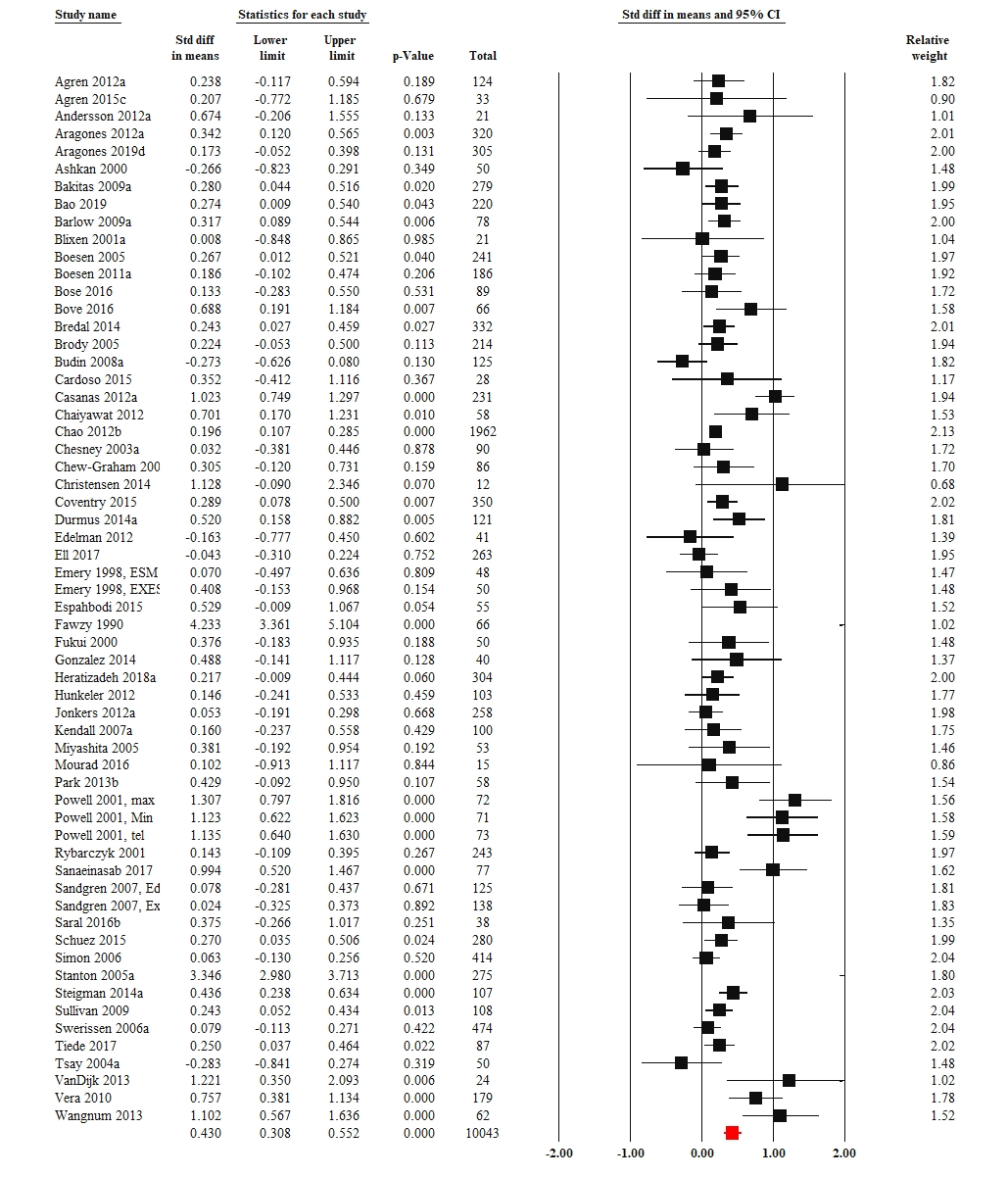


Supplementary Figure 11: Forest plot for quality of life- mental domain

Supplementary Figure 12: Forest plot for physical health related quality of life


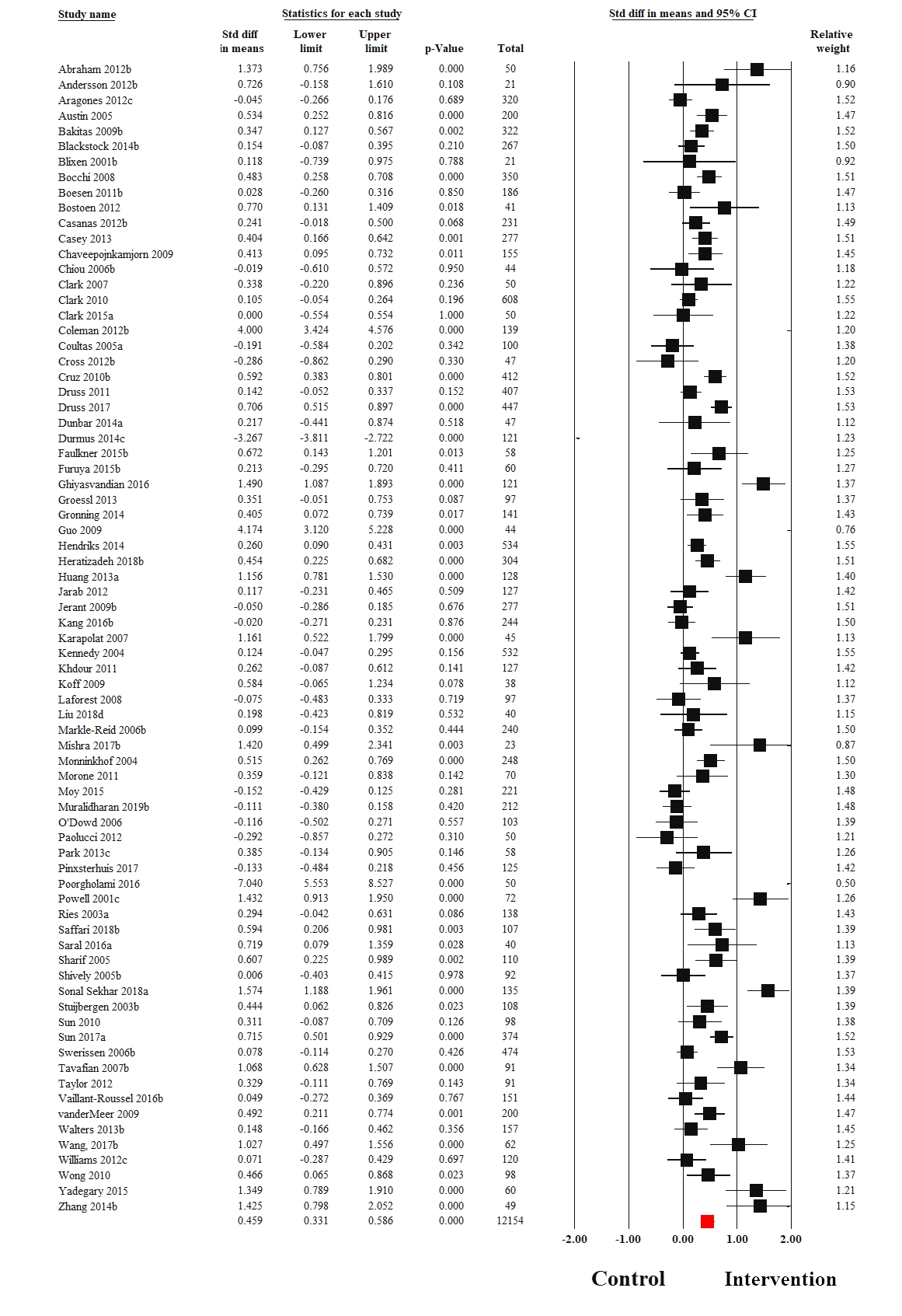


Supplementary Figure 13: Forest plot for biomedical outcomes

Supplementary figure 14: Funnel plot for social functioning

Supplementary figure 15: Funnel plot for adherence to treatment regimen

figure 7:

Supplementary figure 16: Funnel plot for health related knowledge

Supplementary figure 17: Funnel plot for self-efficacy

Supplementary figure 18: Funnel plot for psychological health

Supplementary figure 19: Funnel plot for mental health related quality of life

Supplementary figure 20: Funnel plot for physical health related quality of life

Supplementary table 1: Moderator analyses for outcome of social functioning

| **Group** | **Number Studies** | **Point estimate** | **Standard error** | **Variance** | **Lower limit** | **Upper limit** | **Q-value** | **df (Q)** | **P-value** | **I-squared** |
| --- | --- | --- | --- | --- | --- | --- | --- | --- | --- | --- |
| **System** | | | | | | | | | | |
| **Metabolic** | 1 | 2.00 | 0.21 | 0.04 | 1.58 | 2.41 | 253.13 | 6 | <0.001 | 0 |
| **Musculoskeletal** | 1 | 0.67 | 0.22 | 0.05 | 0.24 | 1.09 |  |  |  | 0 |
| **Neurology** | 2 | 0.22 | 0.16 | 0.03 | -0.10 | 0.55 |  |  |  | 0 |
| **Oncology** | 1 | 0.38 | 0.14 | 0.02 | 0.09 | 0.66 |  |  |  | 0 |
| **Psychiatry** | 1 | -0.04 | 0.13 | 0.02 | -0.29 | 0.21 |  |  |  | 0 |
| **Renal** | 1 | 1.94 | 0.34 | 0.12 | 1.26 | 2.61 |  |  |  | 0 |
| **Rheumatology** | 1 | 4.98 | 0.34 | 0.12 | 4.30 | 5.65 |  |  |  | 0 |
| **Delivery agent** | | | | | | | | | | |
| **Allied health workers** | 3 | 2.41 | 0.85 | 0.72 | 0.75 | 4.08 | 4.02 | 3.00 | 0.26 | 98.80 |
| **Doctors** | 1 | -0.04 | 1.46 | 2.12 | -2.90 | 2.81 |  |  |  | 0.00 |
| **Multidisciplinary** | 3 | 0.34 | 0.85 | 0.72 | -1.32 | 2.01 |  |  |  | 31.65 |
| **Research team** | 1 | 1.94 | 1.49 | 2.22 | -0.98 | 4.86 |  |  |  | 0.00 |
| **Format of delivery** | | | | | | | | | | |
| **Group** | 4 | 1.45 | 0.70 | 0.49 | 0.08 | 2.82 | 1.01 | 3.00 | 0.80 | 98.42 |
| **Individual** | 1 | 1.94 | 1.42 | 2.02 | -0.85 | 4.72 |  |  |  | 0.00 |
| **Mixed** | 2 | 1.13 | 0.99 | 0.97 | -0.80 | 3.07 |  |  |  | 97.24 |
| **Telephone** | 1 | 0.09 | 1.41 | 2.00 | -2.69 | 2.86 |  |  |  | 0.00 |

Supplementary table 2: Moderator analyses for outcome of adherence

| **Group** | **Number Studies** | **Point estimate** | **Standard error** | **Variance** | **Lower limit** | **Upper limit** | **Q-value** | **df (Q)** | **P-value** | **I-squared** |
| --- | --- | --- | --- | --- | --- | --- | --- | --- | --- | --- |
| **System** | | | | | | | | | | |
| **Cardiovascular** | 4 | 1.50 | 0.28 | 0.08 | 0.95 | 2.04 | 16.24 | 11.00 | 0.13 | 93.33 |
| **Gastroenterology** | 2 | 0.74 | 0.42 | 0.17 | -0.08 | 1.56 |  |  |  | 73.05 |
| **Infectious** | 2 | 0.35 | 0.38 | 0.14 | -0.38 | 1.09 |  |  |  | 0.00 |
| **Metabolic** | 2 | 0.31 | 0.39 | 0.15 | -0.44 | 1.07 |  |  |  | 0.00 |
| **Multiple** | 1 | 0.49 | 0.54 | 0.29 | -0.57 | 1.54 |  |  |  | 0.00 |
| **Neurology** | 1 | 1.70 | 0.63 | 0.39 | 0.47 | 2.93 |  |  |  | 0.00 |
| **Oncology** | 1 | 0.80 | 0.60 | 0.35 | -0.37 | 1.96 |  |  |  | 0.00 |
| **Pharmacology** | 3 | 0.27 | 0.33 | 0.11 | -0.38 | 0.91 |  |  |  | 88.17 |
| **Psychiatry** | 4 | 0.74 | 0.29 | 0.09 | 0.17 | 1.31 |  |  |  | 74.74 |
| **Respiratory** | 2 | 0.45 | 0.38 | 0.15 | -0.29 | 1.20 |  |  |  | 85.80 |
| **Rheumatology** | 1 | 0.99 | 0.54 | 0.30 | -0.08 | 2.05 |  |  |  | 0.00 |
| **Urology** | 1 | 0.10 | 0.58 | 0.33 | -1.03 | 1.23 |  |  |  | 0.00 |
| **Delivery agent** | | | | | | | | | | |
| **Allied health workers** | 17 | 0.70 | 0.17 | 0.03 | 0.37 | 1.04 | 0.89 | 2.00 | 0.64 | 87.81 |
| **Doctors** | 1 | 0.21 | 0.71 | 0.50 | -1.18 | 1.60 |  |  |  | 0.00 |
| **Multidisciplinary team** | 6 | 0.89 | 0.28 | 0.08 | 0.34 | 1.44 |  |  |  | 95.63 |
| **Mode of delivery** | | | | | | | | | | |
| **Group** | 2 | 0.68 | 0.52 | 0.27 | -0.34 | 1.70 | 0.82 | 4.00 | 0.94 | 68.85 |
| **Individual** | 11 | 0.83 | 0.22 | 0.05 | 0.40 | 1.27 |  |  |  | 94.55 |
| **Internet** | 2 | 0.56 | 0.53 | 0.28 | -0.48 | 1.61 |  |  |  | 14.94 |
| **Mixed** | 2 | 0.94 | 0.51 | 0.26 | -0.06 | 1.95 |  |  |  | 77.14 |
| **Telephone** | 7 | 0.58 | 0.27 | 0.08 | 0.04 | 1.11 |  |  |  | 86.28 |

Supplementary table 3: Moderator analyses for outcome of health knowledge

| **Group** | **Number Studies** | **Point estimate** | **Standard error** | **Variance** | **Lower limit** | **Upper limit** | **Q-value** | **df (Q)** | **P-value** | **I-squared** |
| --- | --- | --- | --- | --- | --- | --- | --- | --- | --- | --- |
| **System** | | | | | | | | | | |
| **Dermatology** | 1 | 0.30 | 1.01 | 1.02 | -1.68 | 2.29 | 7.91 | 10.00 | 0.64 | 0.00 |
| **Gastroenterology** | 2 | 0.68 | 0.72 | 0.52 | -0.74 | 2.09 |  |  |  | 72.69 |
| **Geriatrics** | 1 | 1.66 | 1.01 | 1.02 | -0.32 | 3.64 |  |  |  | 0.00 |
| **Metabolic** | 2 | 2.47 | 0.75 | 0.56 | 1.00 | 3.95 |  |  |  | 98.66 |
| **Musculoskeletal** | 1 | 1.49 | 1.14 | 1.30 | -0.75 | 3.72 |  |  |  | 0.00 |
| **Nephrology** | 1 | 0.48 | 1.02 | 1.05 | -1.53 | 2.48 |  |  |  | 0.00 |
| **oncology** | 1 | 0.25 | 1.01 | 1.03 | -1.74 | 2.24 |  |  |  | 0.00 |
| **Orthopaedics** | 1 | 0.44 | 1.01 | 1.03 | -1.55 | 2.43 |  |  |  | 0.00 |
| **Renal** | 1 | 1.60 | 1.04 | 1.08 | -0.44 | 3.64 |  |  |  | 0.00 |
| **Respiratory** | 3 | 1.13 | 0.60 | 0.36 | -0.06 | 2.31 |  |  |  | 6.99 |
| **Rheumatology** | 2 | 2.04 | 0.73 | 0.53 | 0.62 | 3.47 |  |  |  | 96.61 |
| **Delivery agent** | | | | | | | | | | |
| **Allied health workers** | 6 | 1.02 | 0.39 | 0.15 | 0.25 | 1.78 | 1.87 | 3.00 | 0.60 | 75.77 |
| **Doctors** | 1 | 0.46 | 0.92 | 0.85 | -1.35 | 2.27 |  |  |  | 0.00 |
| **Multidisciplinary** | 8 | 1.52 | 0.33 | 0.11 | 0.87 | 2.17 |  |  |  | 98.66 |
| **research team** | 1 | 0.90 | 0.93 | 0.87 | -0.93 | 2.72 |  |  |  | 0.00 |
| **Mode of delivery** | | | | | | | | | | |
| **Group** | 5 | 1.05 | 0.42 | 0.17 | 0.23 | 1.86 | 6.92 | 4.00 | 0.14 | 98.53 |
| **Individual** | 5 | 0.87 | 0.42 | 0.18 | 0.04 | 1.70 |  |  |  | 87.31 |
| **internet** | 2 | 0.73 | 0.66 | 0.44 | -0.57 | 2.02 |  |  |  | 41.06 |
| **Mixed** | 3 | 2.57 | 0.57 | 0.33 | 1.44 | 3.69 |  |  |  | 95.70 |
| **telephone** | 1 | 1.26 | 0.94 | 0.89 | -0.59 | 3.11 |  |  |  | 0.00 |

Supplementary table 4: Moderator analyses for outcome of self-efficacy

| **Group** | **Number Studies** | **Point estimate** | **Standard error** | **Variance** | **Lower limit** | **Upper limit** | **Q-value** | **df (Q)** | **P-value** | **I-squared** |
| --- | --- | --- | --- | --- | --- | --- | --- | --- | --- | --- |
| **System** | | | | | | | | | | |
| **Cardiovascular** | 10 | 0.48 | 0.14 | 0.02 | 0.21 | 0.75 | 12.05 | 9.00 | 0.21 | 86.22 |
| **Infectious disease** | 1 | 0.32 | 0.43 | 0.19 | -0.53 | 1.17 |  |  |  | 0.00 |
| **Multiple** | 4 | 0.29 | 0.21 | 0.05 | -0.13 | 0.70 |  |  |  | 55.04 |
| **Musculoskeletal** | 1 | 0.00 | 0.42 | 0.18 | -0.83 | 0.83 |  |  |  | 0.00 |
| **Nephrology** | 5 | 0.38 | 0.20 | 0.04 | -0.01 | 0.77 |  |  |  | 0.00 |
| **Neurology** | 5 | 0.44 | 0.20 | 0.04 | 0.05 | 0.83 |  |  |  | 0.00 |
| **Oral** | 1 | 0.32 | 0.44 | 0.19 | -0.55 | 1.18 |  |  |  | 0.00 |
| **Psychiatry** | 3 | 0.13 | 0.25 | 0.06 | -0.35 | 0.62 |  |  |  | 0.00 |
| **Respiratory** | 5 | 0.99 | 0.20 | 0.04 | 0.60 | 1.38 |  |  |  | 93.73 |
| **Rheumatology** | 2 | 0.16 | 0.29 | 0.08 | -0.40 | 0.73 |  |  |  | 0.00 |
| **Delivery agent** | | | | | | | | | | |
| **Allied health workers** | 20 | 0.50 | 0.10 | 0.01 | 0.31 | 0.69 | 1.21 | 3.00 | 0.75 | 83.18 |
| **Multidisciplinary** | 6 | 0.43 | 0.17 | 0.03 | 0.09 | 0.76 |  |  |  | 89.80 |
| **Peers** | 7 | 0.35 | 0.15 | 0.02 | 0.05 | 0.64 |  |  |  | 0.00 |
| **Research staff** | 4 | 0.29 | 0.22 | 0.05 | -0.13 | 0.72 |  |  |  | 0.00 |
| **Delivery format** | | | | | | | | | | |
| **Group** | 18 | 0.45 | 0.10 | 0.01 | 0.26 | 0.63 | 7.82 | 3.00 | 0.05 | 82.94 |
| **Individual** | 9 | 0.24 | 0.14 | 0.02 | -0.03 | 0.51 |  |  |  | 0.00 |
| **Mixed** | 6 | 0.35 | 0.17 | 0.03 | 0.01 | 0.69 |  |  |  | 48.79 |
| **Telephone** | 4 | 0.92 | 0.21 | 0.04 | 0.52 | 1.33 |  |  |  | 93.81 |

Supplementary table 5: Moderator analyses for outcome of psychological health

| **Group** | **Number Studies** | **Point estimate** | **Standard error** | **Variance** | **Lower limit** | **Upper limit** | **Q-value** | **df (Q)** | **P-value** | **I-squared** |
| --- | --- | --- | --- | --- | --- | --- | --- | --- | --- | --- |
| **System** | | | | | | | | | | |
| Cardiovascular | 5 | -0.25 | 0.25 | 0.06 | -0.75 | 0.25 | 6.11 | 11.00 | 0.87 | 0.00 |
| Dermatology | 1 | -0.22 | 0.52 | 0.27 | -1.24 | 0.81 |  |  |  | 0.00 |
| Geriatrics | 1 | -0.20 | 0.51 | 0.26 | -1.20 | 0.81 |  |  |  | 0.00 |
| Infectious disease | 1 | -0.03 | 0.55 | 0.30 | -1.11 | 1.05 |  |  |  | 0.00 |
| Multiple | 5 | -0.13 | 0.23 | 0.05 | -0.59 | 0.33 |  |  |  | 4.73 |
| Musculoskeletal | 4 | -0.44 | 0.32 | 0.10 | -1.06 | 0.19 |  |  |  | 0.00 |
| Nephrology | 3 | -0.17 | 0.32 | 0.10 | -0.80 | 0.46 |  |  |  | 55.17 |
| Neurology | 5 | -0.41 | 0.25 | 0.06 | -0.90 | 0.08 |  |  |  | 67.58 |
| Oncology | 14 | -0.55 | 0.13 | 0.02 | -0.81 | -0.29 |  |  |  | 95.91 |
| Ophthalmology | 1 | -0.22 | 0.53 | 0.28 | -1.26 | 0.81 |  |  |  | 0.00 |
| Psychiatry | 15 | -0.58 | 0.14 | 0.02 | -0.86 | -0.31 |  |  |  | 81.47 |
| Respiratory | 5 | -0.31 | 0.26 | 0.07 | -0.81 | 0.20 |  |  |  | 0.00 |
| **Delivery agent** | | | | | | | | | | |
| Allied health workers | 32 | -0.35 | 0.08 | 0.01 | -0.51 | -0.19 | 21.83 | 5.00 | 0.00 | 90.62 |
| Doctors | 3 | -0.69 | 0.30 | 0.09 | -1.29 | -0.10 |  |  |  | 9.32 |
| Internet | 1 | -0.10 | 0.68 | 0.46 | -1.43 | 1.22 |  |  |  | 0.00 |
| Multidisciplinary | 13 | -0.26 | 0.13 | 0.02 | -0.52 | 0.00 |  |  |  | 24.98 |
| Peers | 5 | -0.23 | 0.19 | 0.04 | -0.61 | 0.14 |  |  |  | 39.89 |
| Research staff | 6 | -1.29 | 0.21 | 0.04 | -1.69 | -0.88 |  |  |  | 94.88 |
| **Delivery format** | | | | | | | | | | |
| Group | 26 | -0.42 | 0.09 | 0.01 | -0.61 | -0.24 | 3.40 | 4.00 | 0.49 | 80.55 |
| Individual | 12 | -0.58 | 0.14 | 0.02 | -0.84 | -0.31 |  |  |  | 95.93 |
| Internet | 2 | -0.13 | 0.39 | 0.15 | -0.90 | 0.64 |  |  |  | 0.00 |
| Mixed | 16 | -0.36 | 0.12 | 0.01 | -0.58 | -0.13 |  |  |  | 77.05 |
| Telephone | 4 | -0.16 | 0.23 | 0.05 | -0.61 | 0.29 |  |  |  | 0.00 |

Supplementary table 6: Moderator analyses for outcome of QoL-mental

| **Group** | **Number Studies** | **Point estimate** | **Standard error** | **Variance** | **Lower limit** | **Upper limit** | **Q-value** | **df (Q)** | **P-value** | **I-squared** |
| --- | --- | --- | --- | --- | --- | --- | --- | --- | --- | --- |
| **System** | | | | | | | | | | |
| **Cardiovascular** | 4 | 0.01 | 0.32 | 0.10 | -0.62 | 0.64 | 18.94 | 8.00 | 0.02 | 33.06 |
| **Geriatrics** | 1 | 0.00 | 0.63 | 0.39 | -1.23 | 1.23 |  |  |  | 0.00 |
| **Metabolic** | 2 | 1.55 | 0.46 | 0.21 | 0.65 | 2.45 |  |  |  | 98.05 |
| **Musculoskeletal** | 4 | -0.19 | 0.34 | 0.11 | -0.85 | 0.47 |  |  |  | 97.30 |
| **Neurology** | 3 | 0.22 | 0.39 | 0.15 | -0.53 | 0.98 |  |  |  | 0.00 |
| **Psychiatry** | 5 | 0.26 | 0.28 | 0.08 | -0.30 | 0.81 |  |  |  | 55.96 |
| **Renal** | 1 | 1.86 | 0.70 | 0.49 | 0.49 | 3.24 |  |  |  | 0.00 |
| **Respiratory** | 2 | 0.08 | 0.45 | 0.20 | -0.80 | 0.95 |  |  |  | 0.00 |
| **Rheumatology** | 2 | 1.13 | 0.46 | 0.21 | 0.23 | 2.02 |  |  |  | 91.84 |
| **Delivery agent** | | | | | | | | | | |
| **Allied health workers** | 9 | 0.65 | 0.23 | 0.05 | 0.20 | 1.11 | 11.65 | 4.00 | 0.02 | 94.59 |
| **Doctors** | 3 | -0.76 | 0.39 | 0.16 | -1.53 | 0.01 |  |  |  | 97.51 |
| **Multidisciplinary** | 9 | 0.30 | 0.23 | 0.05 | -0.15 | 0.75 |  |  |  | 75.55 |
| **Peers** | 1 | 0.30 | 0.67 | 0.45 | -1.01 | 1.62 |  |  |  | 0.00 |
| **Research team** | 2 | 1.07 | 0.51 | 0.26 | 0.07 | 2.08 |  |  |  | 92.01 |
| **Delivery format** | | | | | | | | | | |
| **Group** | 13 | 0.20 | 0.21 | 0.05 | -0.22 | 0.62 | 1.63 | 3.00 | 0.65 | 93.68 |
| **Individual** | 4 | 0.61 | 0.39 | 0.15 | -0.14 | 1.37 |  |  |  | 88.29 |
| **Mixed** | 4 | 0.66 | 0.38 | 0.14 | -0.08 | 1.40 |  |  |  | 97.62 |
| **Telephone** | 3 | 0.28 | 0.45 | 0.20 | -0.59 | 1.15 |  |  |  | 35.40 |

Supplementary table 7: Moderator analyses for outcome of QoL-physical health

| **Group** | **Number Studies** | **Point estimate** | **Standard error** | **Variance** | **Lower limit** | **Upper limit** | **Q-value** | **df (Q)** | **P-value** | **I-squared** |
| --- | --- | --- | --- | --- | --- | --- | --- | --- | --- | --- |
| **System** | | | | | | | | | | |
| **Cardiovascular** | 11 | 0.39 | 0.16 | 0.03 | 0.07 | 0.71 | 82.30 | 14.00 | 0.00 | 67.91 |
| **Dermatology** | 4 | 0.90 | 0.28 | 0.08 | 0.36 | 1.44 |  |  |  | 82.62 |
| **Gastroenterology** | 4 | 0.50 | 0.27 | 0.08 | -0.04 | 1.03 |  |  |  | 93.15 |
| **Geriatrics** | 1 | 0.10 | 0.53 | 0.28 | -0.93 | 1.13 |  |  |  | 0.00 |
| **Infectious disease** | 3 | 0.18 | 0.33 | 0.11 | -0.47 | 0.83 |  |  |  | 0.00 |
| **Metabolic** | 3 | 0.68 | 0.31 | 0.10 | 0.07 | 1.29 |  |  |  | 94.13 |
| **Multiple** | 5 | 0.16 | 0.24 | 0.06 | -0.31 | 0.63 |  |  |  | 90.42 |
| **Musculoskeletal** | 8 | 0.14 | 0.21 | 0.04 | -0.27 | 0.56 |  |  |  | 96.39 |
| **Nephrology** | 1 | 7.04 | 0.91 | 0.84 | 5.25 | 8.83 |  |  |  | 0.00 |
| **Neurology** | 5 | 1.12 | 0.27 | 0.07 | 0.60 | 1.65 |  |  |  | 92.03 |
| **Oncology** | 2 | 0.19 | 0.37 | 0.14 | -0.54 | 0.92 |  |  |  | 66.54 |
| **Psychiatry** | 11 | 0.42 | 0.17 | 0.03 | 0.09 | 0.75 |  |  |  | 88.05 |
| **Renal** | 2 | 0.88 | 0.40 | 0.16 | 0.09 | 1.67 |  |  |  | 82.86 |
| **Respiratory** | 19 | 0.24 | 0.13 | 0.02 | -0.01 | 0.49 |  |  |  | 56.61 |
| **Rheumatology** | 3 | 1.54 | 0.32 | 0.10 | 0.91 | 2.17 |  |  |  | 98.34 |
| **Delivery agent** | | | | | | | | | | |
| **Allied health workers** | 35 | 0.52 | 0.09 | 0.01 | 0.34 | 0.71 | 23.75 | 5.00 | 0.00 | 87.96 |
| **Doctors** | 5 | -0.30 | 0.24 | 0.06 | -0.78 | 0.17 |  |  |  | 97.74 |
| **Internet** | 1 | -0.15 | 0.53 | 0.28 | -1.19 | 0.89 |  |  |  | 0.00 |
| **Multidisciplinary** | 21 | 0.48 | 0.12 | 0.01 | 0.24 | 0.71 |  |  |  | 86.48 |
| **Peers** | 6 | -0.01 | 0.22 | 0.05 | -0.44 | 0.41 |  |  |  | 3.87 |
| **Research staff** | 8 | 1.07 | 0.21 | 0.04 | 0.66 | 1.48 |  |  |  | 93.14 |
| **Delivery format** | | | | | | | | | | |
| **Group** | 30 | 0.44 | 0.10 | 0.01 | 0.28 | 0.65 | 5.31 | 5.00 | 0.38 | 93.60 |
| **Individual** | 16 | 0.49 | 0.15 | 0.02 | 0.20 | 0.78 |  |  |  | 89.18 |
| **Internet** | 2 | -0.21 | 0.41 | 0.17 | -1.02 | 0.60 |  |  |  | 0.00 |
| **Mixed** | 22 | 0.60 | 0.12 | 0.02 | 0.35 | 0.84 |  |  |  | 87.51 |
| **phone** | 1 | 0.15 | 0.56 | 0.32 | -0.96 | 1.25 |  |  |  | 0.00 |
| **Telephone** | 4 | 0.17 | 0.29 | 0.09 | -0.40 | 0.74 |  |  |  | 0.00 |

Supplementary table 8: Moderator analyses for outcome of biomedical indicators

| **Group** | **Number Studies** | **Point estimate** | **Standard error** | **Variance** | **Lower limit** | **Upper limit** | **Q-value** | **df (Q)** | **P-value** | **I-squared** |
| --- | --- | --- | --- | --- | --- | --- | --- | --- | --- | --- |
| **Outcomes** | | | | | | | | | | |
| Biological rhythm | 1 | 0.66 | 0.52 | 0.27 | -0.35 | 1.68 | 68.27 | 21.00 | <0.005 | 0.00 |
| Blood pressure | 21 | 0.17 | 0.08 | 0.01 | 0.01 | 0.33 |  |  |  | 81.25 |
| BMI | 3 | 0.36 | 0.22 | 0.05 | -0.07 | 0.79 |  |  |  | 0.00 |
| Body weight | 8 | 0.43 | 0.14 | 0.02 | 0.16 | 0.70 |  |  |  | 84.45 |
| Cardiovascular risk | 3 | 0.30 | 0.21 | 0.04 | -0.11 | 0.71 |  |  |  | 88.86 |
| Cytokine levels | 1 | 0.73 | 0.46 | 0.21 | -0.16 | 1.62 |  |  |  | 0.00 |
| eGFR | 3 | 0.91 | 0.21 | 0.04 | 0.50 | 1.32 |  |  |  | 60.49 |
| FEV1 | 1 | 0.13 | 0.37 | 0.14 | -0.60 | 0.87 |  |  |  | 0.00 |
| Gingival index | 1 | 1.76 | 0.40 | 0.16 | 0.98 | 2.54 |  |  |  | 0.00 |
| INR | 1 | 0.18 | 0.37 | 0.13 | -0.54 | 0.90 |  |  |  | 0.00 |
| Interidalytic weight gain | 2 | 0.29 | 0.27 | 0.07 | -0.25 | 0.82 |  |  |  | 0.00 |
| Serum albumin | 1 | 0.09 | 0.36 | 0.13 | -0.63 | 0.80 |  |  |  | 0.00 |
| Serum Calcium and Phosphorus product | 3 | 0.13 | 0.22 | 0.05 | -0.31 | 0.56 |  |  |  | 0.00 |
| Serum cholesterol | 7 | 0.63 | 0.15 | 0.02 | 0.33 | 0.92 |  |  |  | 97.39 |
| Serum creatine | 1 | 0.04 | 0.47 | 0.22 | -0.88 | 0.95 |  |  |  | 0.00 |
| Serum glucose | 2 | 0.92 | 0.26 | 0.07 | 0.41 | 1.44 |  |  |  | 74.04 |
| Serum HbA1c | 32 | 0.28 | 0.07 | 0.00 | 0.14 | 0.41 |  |  |  | 85.99 |
| Serum phosphate levels | 6 | 0.36 | 0.16 | 0.02 | 0.06 | 0.67 |  |  |  | 34.76 |
| Sodium excretion rate | 1 | 0.83 | 0.38 | 0.14 | 0.09 | 1.57 |  |  |  | 0.00 |
| UKPDS risk | 1 | -0.14 | 0.36 | 0.13 | -0.84 | 0.56 |  |  |  | 0.00 |
| Urine albumin | 1 | -1.55 | 0.36 | 0.13 | -2.26 | -0.84 |  |  |  | 0.00 |
| Waist circumference | 1 | 0.40 | 0.36 | 0.13 | -0.32 | 1.11 |  |  |  | 0.00 |
| Overall | 101 | 0.36 | 0.09 | 0.01 | 0.18 | 0.54 |  |  |  |  |
